# Human Locomotion

**Published:** 1874-11

**Authors:** 


### HUMAN LOCOMOTION.

The movements executed by animals in transporting themselves from place to place have long engaged the attention of observers; and, as animals which travel on the land are more easily got at than those which freqnent the sea and the air, it is the motions of such that we know most about. Yet much remains to be learned of the modes of progression of even the most familiar of these; and not a little probably will have to be unlearned that recent investigations have shown to be erroneous.

At first sight, the operations of walking and running, as displayed by both two-legged and four-legged creatures, may appear simple enough, but all attempts to analyze and explain them have shown that in reality they are very complex, so that there has arisen a wide diversity of opinion concerning their real nature. These disagreements among the investigators of the subject can only be accounted for on the principle of the insufficiency of the means at their command for complete investigation; and the confusion has been further increased by the difficulty of expressing in words the rhythm, duration, and phases of the rapid and complex movements involved.

Prof. E. J. Marey, of the College of France, a skillful physiologist, and the inventor of the various delicate mechanical appliances for tracing and registering obscure animal motions, has contributed to the "International Scientific Series" a work entitled "Animal Mechanism," in which the subject of terrestrial locomotion, as typified in man and in the horse, is fully treated. Prof. Marey has devised an apparatus which, applied to the extremities of a moving animal, enables each limb to write out a description, or make a picture of its own actions, so that the duration and phases of its movements, its periods of rest, and the relations of these to the corresponding features in the motions of the other limbs, may be seen at a glance. A description of this apparatus, and its mode of operation, with the results that it gives when applied to man, will first engage our attention.—*Popular Science Monthly for November.*

